# Rupture hémorragique d’une malformation arterioveineuse géante hez un sujet jeune à Ouagadougou

**DOI:** 10.11604/pamj.2018.29.38.12253

**Published:** 2018-01-17

**Authors:** Anselme Alfred Dabilgou, Julie Marie Adeline Kyelem

**Affiliations:** 1 Department of Neurology, Teaching Hospital Yalgado Ouedraogo of Ouagadougou

**Keywords:** Hémorragie cérébrale, thrombose du sinus caverneux, neurologie, Cerebral hemorrhage, thrombosis of the cavernous sinuses, neurology

## Abstract

Vascular arteriovenous malformations (VAM) are aggressive vascular diseases associated with increased haemorrhagic risk in terms of morbidity and mortality. Vascular arteriovenous malformations are usually sporadic. The main symptom of VAM is hemorrhage, in 50% of cases. We here report the case of a 28 year old patient, hospitalized with right sided motor deficit of acute onset associated with consciousness disorder in the Department of Neurology at the Yalgado Ouédraogo University Hospital Center. The patient had a history of chronic headaches treated with self-medication and of right Bravais-Jacksonian seizures. Neurological examination, performed on admission, showed proportional right hemiplegia, Broca's aphasia, left eye pain associated with ptosis, left chemosis and febrile meningeal syndrome with a temperature at 40°C. On admission the NIHSS score was 21/42 and the ICH score was 2. Emergency cerebral CT scan showed cerebral hemorrhage in the basal ganglia and in the brainstem, intra-ventricular flood and basal cisterns. Blood count showed leukocytosis at 13 200/mm^3^ with granulocytic predominance (72.6%). Chest X-ray showed right alveolar pneumopathy. Tuberculin intradermal reaction (IDR) was positive to 17mm. Cerebral angioscanner, performed on day 24, showed arteriovenous malformation of the middle cerebral arteries and of the posterior vertebral arteries as well as thrombosis of the cavernous sinuses.

## Image en médecine

Les malformations vasculaires artériole-veineuses sont des pathologies vasculaires agressives présentant un risque hémorragique lourd de conséquence en termes de morbi-mortalité. Les MAV sont en majorité sporadiques. Le mode de révélation de la MAV est une hémorragie dans 50% des cas. A travers cet article, nous rapportons l'observation clinique d'un patient de 28 ans, hospitalisé dans le service de neurologie du CHU Yalgado Ouédraogo pour déficit moteur de l'hémicorps droit d'installation brutale accompagné de trouble de la conscience. Dans les antécédents, on notait des céphalées chroniques traitées par automédication et des crises épileptiques bravais jacksoniennes droites. L'examen neurologique à l'entrée retrouvait une hémiplégie droite proportionnelle, une aphasie de Broca, une ophtalmologie gauche douloureuse avec ptosis, un chémosis gauche et un syndrome méningé fébrile avec une fièvre à 40°C. Le score NIHSS était à 21/42 et un score ICH à 2 à l'entrée. Le scanner cérébral en urgence montrait une hémorragie cérébrale des noyaux gris centraux et du tronc cérébral, une inondation intra-ventriculaire et des citernes de la base. L'hémogramme notait une hyperleucocytose à 13 200/mm^3^ à prédominance granulocytaire (72.6%). La radiographie pulmonaire montrait une pneumopathie droite de type alvéolaire. L'intradermo-reaction (IDR) à la tuberculine était positive à 17mm. L'angioscanner cérébrale à distance (J24) retrouvait une malformation arterio-veineuse des artères cérébrales moyennes et vertébrales postérieures et une thrombose du sinus caverneux.

**Figure 1:**
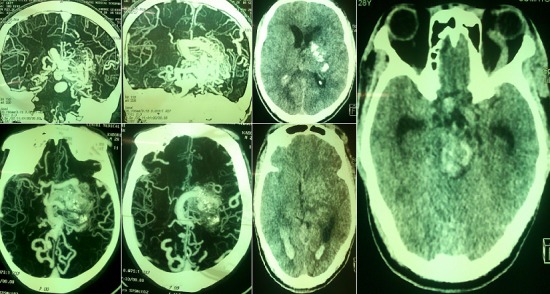
Malformation géante

